# The Innate Immune Database (IIDB)

**DOI:** 10.1186/1471-2172-9-7

**Published:** 2008-03-05

**Authors:** Martin Korb, Aistair G Rust, Vesteinn Thorsson, Christophe Battail, Bin Li, Daehee Hwang, Kathleen A Kennedy, Jared C Roach, Carrie M Rosenberger, Mark Gilchrist, Daniel Zak, Carrie Johnson, Bruz Marzolf, Alan Aderem, Ilya Shmulevich, Hamid Bolouri

**Affiliations:** 1 Institute for Systems Biology, 1441 North 34thStreet, Seattle, Washington 98103-8904, USA; 2 Institute Curie, 26 rue d'Ulm 75248 Paris cedex 05, France; 3 Department of Chemical Engineering, Pohang University of Science and Technology, San 31, Hoja-Dong, Nam-Gu, Pohang, Kyungbuk, 790-784, Republic of Korea

## Abstract

**Background:**

As part of a National Institute of Allergy and Infectious Diseases funded collaborative project, we have performed over 150 microarray experiments measuring the response of C57/BL6 mouse bone marrow macrophages to toll-like receptor stimuli. These microarray expression profiles are available freely from our project web site . Here, we report the development of a database of computationally predicted transcription factor binding sites and related genomic features for a set of over 2000 murine immune genes of interest. Our database, which includes microarray co-expression clusters and a host of web-based query, analysis and visualization facilities, is available freely via the internet. It provides a broad resource to the research community, and a stepping stone towards the delineation of the network of transcriptional regulatory interactions underlying the integrated response of macrophages to pathogens.

**Description:**

We constructed a database indexed on genes and annotations of the immediate surrounding genomic regions. To facilitate both gene-specific and systems biology oriented research, our database provides the means to analyze individual genes or an entire genomic locus. Although our focus to-date has been on mammalian toll-like receptor signaling pathways, our database structure is not limited to this subject, and is intended to be broadly applicable to immunology. By focusing on selected immune-active genes, we were able to perform computationally intensive expression and sequence analyses that would currently be prohibitive if applied to the entire genome. Using six complementary computational algorithms and methodologies, we identified transcription factor binding sites based on the Position Weight Matrices available in TRANSFAC. For one example transcription factor (ATF3) for which experimental data is available, over 50% of our predicted binding sites coincide with genome-wide chromatin immnuopreciptation (ChIP-chip) results. Our database can be interrogated via a web interface. Genomic annotations and binding site predictions can be automatically viewed with a customized version of the Argo genome browser.

**Conclusion:**

We present the Innate Immune Database (IIDB) as a community resource for immunologists interested in gene regulatory systems underlying innate responses to pathogens. The database website can be freely accessed at .

## Background

Extensive transcriptional regulation underlies macrophage responses to toll-like receptor (TLR) signaling [1]. Differential transcriptional activity in response to TLR signaling tailors macrophage responses to different pathogens [2-8]. See [9] for a review. In spite of recent achievements [10-12], the cost and difficulty of comprehensive experimental identification of transcription factor binding sites (e.g. using ChIP-chip technology [13]) continues to be high. Computational methods can aid these efforts by predicting potential transcription factor-DNA interactions in response to pathogens. The information necessary for comprehensive prediction of transcription factor binding sites (TFBSs) on large numbers of genes is currently dispersed in publications [14-18] and across various databases such as ENSEMBL [19], GenBank [20], TRANSFAC [21], JASPAR [22], cisRED [23], and EPD [24].

While there are other examples of mouse genome annotation databases and websites [25,26], in this paper, we present a database that is specifically focused on mouse innate immunity genes and their predicted regulatory interactions. The Innate Immune Database (IIDB) contains annotations for over 2000 genes, including over 1600 TLR-responsive genes, and additional genes considered of importance to innate immune responses. We have annotated these genes with data from over 150 microarray experiments, the ENSEMBL database, the database resources of the National Center for Biotechnology Information (NCBI), computationally predicted TFBSs, predicted *cis- *regulatory modules, evolutionary conserved regulatory sequences, DNase hypersensitive sites, co-expression clusters, and genome-wide chromatin immunoprecipitation (ChIP-chip) data. For each gene, we have analyzed the sequence from at least 20 kb upstream to at least 10 kb downstream of the predicted transcription end site, including all exons and introns. In addition to regulatory predictions, annotations for exon/intron boundaries, CpG islands, repeats, Affymetrix Microarray Expression probes are included. Users can interactively interrogate IIDB using a web interface. Search results are mapped to the genome sequence. Standard text-based files are created ('.gff2' and '.gff3') and can be explored visually using the web-enabled Argo genome browser [27].

## Construction and Content

IIDB uses the MYSQL relational database system to store, retrieve and manage the data. The web interface between the user and IIDB is coded in PERL/CGI. Initially, the database was populated with a set of 1670 genes differentially regulated in response to LPS. We subsequently added a selection of approximately 400 other genes suspected or recognized to be of importance to the innate immune system. Approved (data curator) users can easily upload additional genes for analysis by providing the Entrez GeneID via the web interface. The uploading validation and annotation process is shown in Additional File 1. Uploaded genes are automatically annotated and made available via the web interface, usually within hours. At this time, we limit web based user submission to 300 kb, although longer loci can be handled by special request.

### Sequence Coordinates and Gene Mapping

To accommodate the needs of our multiple ongoing experimental projects and users, IIDB provides annotations based on both the UCSC mouse genome version mm5 [28] and the ENSEMBL mouse 29e build [19]. The two gene maps differ in the number of predicted genes and other details. We built two distinct gene coordinate maps for IIDB, one based on UCSC data, and the other based on ENSEMBL. Gene loci are labeled by the gene name and corresponding Entrez geneID. A chromosome locus may contain more than one gene. Therefore IIDB allows multiple labels to be associated with a single locus. Locus coordinates were used to search the ENSEMBL database for repeats, CpG islands, and Affymetrix Microarray Expression ProbeSets (from the Mouse Genome 430 2.0 Array).

### Predicted Individual Transcription Factor Binding Sites

We annotated the genomic sequence of all the genes in our database with each of the 360 mouse-specific individual TRANSFAC (Professional edition 8.3, [21]) Position Weight Matrices (PWM) using the MotifLocator algorithm[29]. Scanning was performed on both the positive and negative strands. To assess the statistical significance of MotifLocator scores and to set selection thresholds, we evaluated MotifLocator scores on 200 kb of shuffled sequences from upstream regions of approximately 100 immune related genes. The randomization procedure was repeated six times for a total of 1.2 × 10^6 ^random scores per matrix. For each PWM, MotifLocator scores for the true sequence were converted to p-values by comparison to the score distribution for the same PWM on the randomized sequence. Based on this analysis we generated matrix scan datasets with p-values less than 1 × 10^-3^, 5 × 10^-4 ^and 1 × 10^-4^.

### Summary Presentation of Binding Sites for Similar Factors

Display of all individual putative TFBSs can produce cluttered visualizations. To provide an alternative representation, avoiding presentation of overlapping hits of identical or similar matrices, we grouped the 360 individual TRANSFAC matrices into 67 matrix families and 76 individual matrices. First, we computationally grouped TRANSFAC matrices whose identifiers correspond to the same transcription factor into a single matrix group. For example, AHR_Q5, AHR_01 both identify the Ahr transcription factor. Next, we computationally combined our matrix groups with TRANSFAC matrices whose identifiers indicated that they belong to the same transcription factor family. For example, matrices for MYC_Q2, EBOX_Q6, and MYCMAX_02 were combined. Finally, we hand curated the groups and remaining single matrices to ensure against computational false positives or false negatives. Any matrices not assigned into a group by the above procedure were retained as individual matrices.

To remove redundant predictions, overlapping hits from the same PWM group were collapsed into a single predictive hit if the two predicted TFBS overlapped by at least half of the length of the 5' matrix (|start_matrix1 _- start_matrix2_| <= length_matrix1_/2). If the neighboring matrix did not satisfy this condition, it was marked as a distinct TFBS. The same algorithm was used to combine identical overlapping single matrix hits (see Additional File 2). This methodology significantly reduces the number of predicted TFBS and avoids visualization clutter. For example, combining individual matrices into matrix families reduced the number of predicted transcription factor binding sites from 2500 to 1795 at a p-value < 1 × 10^-4 ^over the 54 Kbp sequence analyzed for the *Interleukin 12b *(*IL12b*) locus.

### Spatial Clusters of TFBS

Transcription factors often bind in close proximity of each other within a *cis-*regulatory module [25,26,30]. We used the COBALT Clustering algorithm CCA (Battail C, Hwang D, Rust A, et al, manuscript in preparation) to identify statistically significant spatial clusters of matrix hits (cluster p-value ≤ 10^-2^). Briefly, the algorithm detects clusters of TFBS hits on a DNA regulatory region previously scanned by a library of matrices (e.g., from TRANSFAC or JASPAR). A "maximum cluster size" parameter limits the sequence length over which a cluster can extend. We chose 500 bp for this parameter based on typical lengths of known *cis*-regulatory modules in animal genes [30]. A score is assigned to each cluster based on the motif scores that comprise the cluster and the spatial density of motifs. A "maximum motif overlap" parameter sets up the maximum percentage of overlap for two motifs to be considered individually. A list of cluster scores generated is compared to a list of cluster scores generated from a background DNA sequence. This comparison is then used to assign a p-value to each motif cluster according to its significance.

### Conserved Human/Mouse/Rat/Dog Promoter Sequences

IIDB includes a catalog of over 26000 conserved human/rat/mouse and dog promoter sequences identified by Xie et al. [14]. To map the reported conserved sequences to the promoter region of the genes in our database, we used a simple nucleotide search algorithm accepting only exact matches to the published sequences. Since Xie et al only investigated human promoters up to 2 Kb upstream of the transcription start site, we disregarded all mouse sequence matches located further than 3 kb upstream and 500 bp downstream of the transcription start site. In addition, we disregarded matches that were less than 100 nucleotides in length. The original list of conserved genomic sequences can be accessed at [31]. As the state of the art and the data evolve, we anticipate periodic updates to annotations based on phylogenetic conservation.

### Mouse Homologs of Human DNase Hypersensitive Sites

DNase hypersensitive (HS) sites can help identify the location of *cis-*regulatory regions on DNA [32,33]. We used the multicross species DNase HS site mapping information as reported by Crawford et al [31] to create the chromosome coordinates for over 16500 possible mouse DNase HS sites. Briefly, we used the human DNase coordinate information, flanked by an additional 10 bp 5' and 3', to define a human HS sequence. This sequence was used as the input to the ENSEMBL Compara36 database to identify the coordinates of the matching mouse sequences. Only the top 12 mouse hits per human DNase HS sequence slice are used to populate our database. The original map of human DNase HS can be found at [34].

### ChIP-chip Data

To demonstrate the ease of integrating additional data into IIDB, we have included data from a genome wide chromatin immunoprecipitation assay, employing a custom Affymetrix oligonucleotide array [35]. This array contains densely-tiled 25-mer oligonucleotide sequences designed to interrogate almost all of the C57/BL6 mouse macrophage genes in IIDB. The raw data was processed with quantile normalization [36,37], then filtered with a sliding window median filter to identify putative binding sites [37]. IIDB includes both the tiling probe locations on all genes, and also the locations of statistically significant binding hits.

## Utility and Discussion

### User Interface

**Figure 1:**
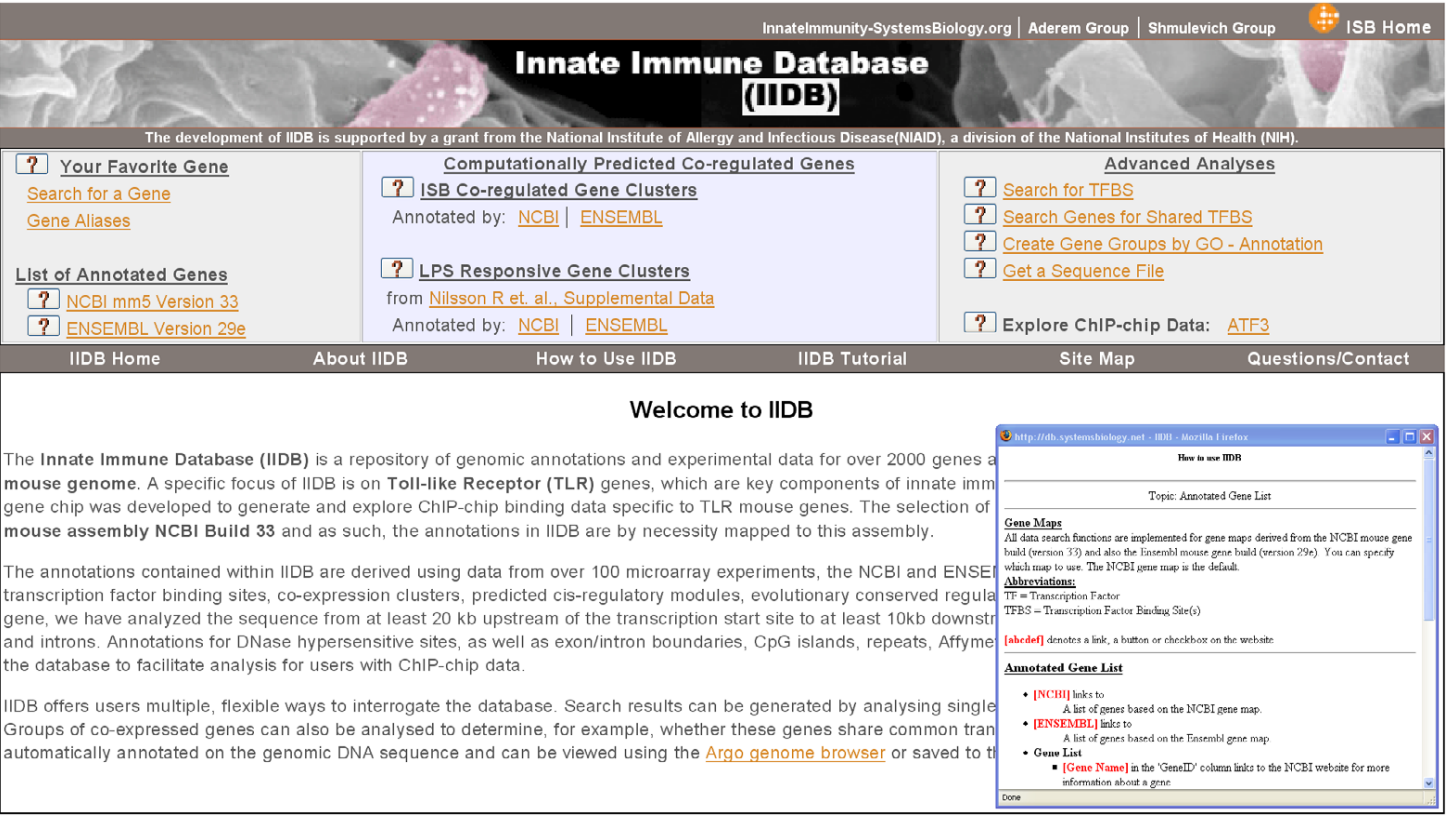
**Snapshot of the IIDB web site entry page**. The entry page includes links to user guides ('How to Use IIDB' and 'IIDB Tutorial'), and links to allow the searching and visualization of IIDB content in a variety of ways, as described in the main text. We also provide links to 3^rd ^party data and software used by IIDB. Clicking the '**?**' symbol located to the left of each menu item pops open a help page explaining how to use that menu item (**Inset**).

The user can interactively interrogate annotated genes using either the UCSC database version mm5 gene map or the ENSEMBL mouse 29e gene map. All interactions are via a web interface. Searches can be performed by providing a common name (e.g. *IL12b*), Entrez geneid (e.g. 16160), Refseq (e.g. NM_008352), or a chromosomal location (e.g. chr:11 44019798–44073744). See Fig. 1 for a snapshot of the IIDB entry page. Using a set of check boxes, users can select single genes or genes co-expressed under TLR stimulation, and search the gene(s) for putative binding sites of all factors in TRANSFAC, or the predicted binding sites of specified transcription factors. Additional check boxes allow the user to merge TFBS hits into families of binding sites with similar weight matrices; identify spatially clustered TFBS's, find TFBS hits shared by a group of genes; and view DNase HS sites, evolutionary conserved regions, and ChIP-chip hits. Additionally, the locations of exons/introns, CpG islands and repeats, and Affymetrix expression and tiling probes are available for viewing in the result set.

Users can filter all TFBS predictions at any of three p-values: 1 × 10^-3^, 5 × 10^-4 ^and 1 × 10^-4^. The more stringent p-values greatly reduce the occurrence of background (non-significant) matrix hits but may miss some true binding sites. Taking the *IL12b *gene as an example, we analyzed 54 kbp of sequence. The above methodology reduced the number of predicted TFBS from 15810 hits at a p-value threshold of 10^-3 ^to 1795 at a p-value threshold of 10^-4^. We selected 10^-4 ^as the most stringent p-value threshold because filtering at this level still allows exact matches to 67 out of 80 (84%) of known (TRANSFAC validated) TFBS's in our data set.

The results of the user's selections are temporarily stored in '.gff2' and '.gff3' file formats on our server and can be downloaded, saved to the user's computer and exported to other applications. Alternatively, the user can click on a link on the IIDB web page to invoke the Java Web Start enabled genome browser Argo (version 1.0.21, [27]). This facility relieves the user from the need to download/install any software.

For greater stringency of results, IIDB allows the user to filter transcription factor binding site predictions in several different ways as listed below:

#### Single Gene Analysis

All DNA sequences in our database have been annotated for exon/intron boundaries, repeats, CpG islands and Affymetrix probes. All sequences were scanned using the 360 TRANSFAC mouse-specific PWMs (Professional edition 8.3, [21]). Each gene in IIDB is marked with a set of character symbols indicating the availability of different kinds of data for that gene.

**Figure 2:**
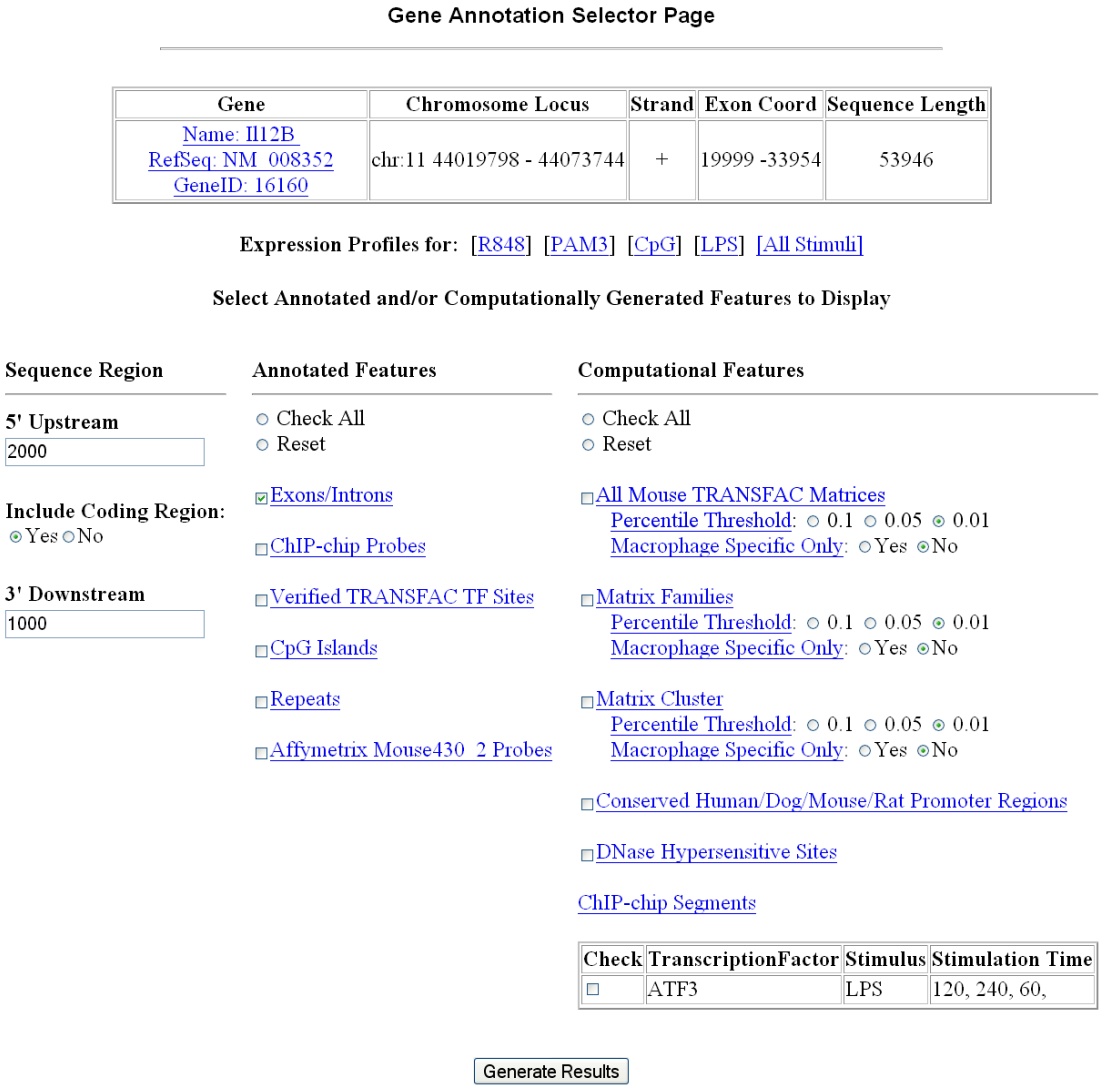
**Exploring an annotated gene sequence**. The user can choose the size of 5' upstream promoter and 3' downstream regions to search, and whether to include features identified within a gene's coding region. A link is provided to all available microarray expression profiles. The user can choose one or all features for viewing, including the list of putative regulatory transcription factors and significance thresholds. Each feature name is linked to additional pages with more information about that particular feature.

Users can query a gene for some or all information available in IIDB. The strand, sequence length, and exon coordinates are provided. Based on this information, the user can limit the search region by specifying 5' upstream and 3' downstream regions (Fig. 2). Furthermore, we provide microarray expression data for genes differentially expressed in response to TLR stimulation (see Table 1 for stimulus list).

**Table 1:** Macrophage TLR stimuli used in the experiments underlying IIDB

**Stimulus**	**Description**
CpG	Unmethylated CpG motif (cytosine and guanine separated by a phosphate) bacterial DNA (TLR9-specific stimulant)
LPS	Lipopolysaccharide (component of the cell membrane of Gram-negative bacteria), TLR4-specific stimulant
PAM_2_CSK	Synthetic diacylated lipoprotein, TLR2/6 stimulant
PAM_3_CSK	Synthetic triacylated lipopeptide, TLR 2/1 stimulant
Poly(I:C)	Polyriboinosinic polyribocytidylic acid (TLR3-specific stimulant)
R848	Synthetic imidazoquinoline resiquimod, TLR 7, 8 stimulant

#### Search for TFBS

**Figure 3:**
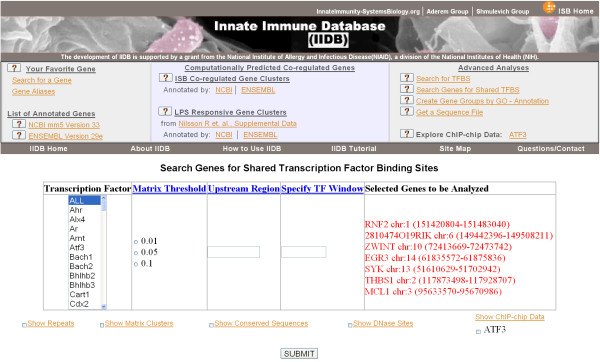
**Searching genes for targets of specific transcription factors**. IIDB provides a list of 268 unique transcription factors in the selection box on the left. The user can select several transcription factors, the p-value, the length of promoter region to explore, and any number of target genes. The target gene column is automatically populated if the user selects genes from a previous page (shown). Otherwise the user can add a comma separated list of gene identifiers or chromosomal locations (not shown). Several additional features can be displayed simultaneously on top of the predicted transcription factor binding sites (selection boxes at bottom). A link to a page which details the upload format for a search file for bulk queries is also provided (not shown).

A total of 143 PWMs representing 268 transcription factors as 67 transcription factor families and 76 single-factor matrices are stored in our database. A user can search for the binding sites of one or several transcription factor families at once, and compare TFBS locations across multiple genes (Fig. 3). Because IIDB generates standard .gff files on-the-fly, the user can select the set of features to view online or export the data to other genome browsers. Also an easy to use file upload facility is available for specifying a large number of transcription factors and their putative target genes in a query. The user is contacted by email when the analysis is complete.

#### Search a Set of Genes for Shared TFBS

Members of a regulatory complex will often have tight spatial constraints on the relative locations of their TFBS. This expectation can be exploited to impose a stringent statistical filter on predicted TFBS's. IIDB users can search a group of potentially co-regulated genes for common transcription factor binding sites within a given distance from each other. The user can choose the genes, set the window size, and specify the length of the regulatory region to be analyzed.

#### Find Spatially Clustered TFBS on a Sequence

Mammalian *cis-*regulatory modules are thought to be typically around 500 bp in length and contain of the order of a dozen or more TFBSs [18,30,38]. This option (available as a tick box at the bottom of each search page) lets the user identify potential *cis-*regulatory regions on gene sequences by searching for statistically unlikely spatial clusters of TFBS's (as compared to TFBS patterns on shuffled sequences) for a range of window sizes.

#### Select Genes by GO Annotation

The user can select a set of IIDB annotated genes based on their common GO annotation [39,40]. The resulting set can then be searched for predicted TFBS or to find a subset of genes with shared TFBS hits.

#### Explore ChIP-chip Data

As an example of additional data integration in IIDB, and to allow evaluation of the accuracy of our TFBS predictions, IIDB includes ChIP-chip data for the ATF3 transcription factor [29]. We plan to include additional ChIP-chip data from other transcription factors as they become available.

#### Argo Genome Browser Display

**Figure 4:**
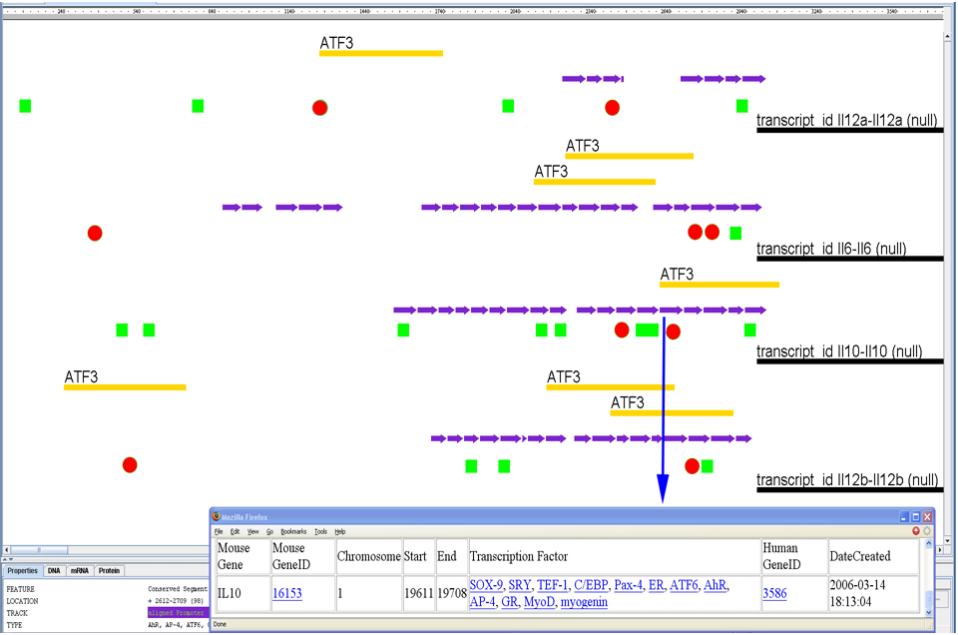
**An example of a web-based Argo multigene display of IIDB search results**. The following genes (IL6, Il10, IL12a, IL12b) were queried for ATF3 predicted and ChIP-chip hits (red circles and orange boxes labeled ATF3, respectively), and predicted NF-κB binding sites (green rectangles) in the proximal promoter regions (+1 to -3000). Evolutionary conserved promoter sequences (purple) are also shown. The predictions are in good agreement with the experimentally ChIP-Chip hits. **Inset: Detail of an Evolutionary Conserved Promoter Region**. By double clicking an evolutionary conserved promoter sequence (purple arrows) a new browser window displays details such as the human ortholog, start and end coordinates, and the human transcription factors associated with this segment (MS Internet Explorer only).

All IIDB search results are color coded in the genome browser view. IIDB maintains a consistent color coding scheme across all gene displays. Within the Argo genome browser, additional specific detail about a particular feature can be displayed by double clicking that feature as shown in Fig. 4 (MS Internet Explorer only). The list of human transcription factors associated with a particular evolutionary conserved promoter sequence can be observed in a new browser window by double clicking the feature in the genome browser. For the 'Matrix Families' feature, the matrix hit with the highest score is displayed. Contributing matrix hits are attributes of the displayed matrix and can be accessed by double clicking on the displayed matrix hit.

#### Help

An extensive help menu is available, as indicated by the '?' symbol next to each link in the navigation bar at the top of each web page. We also provide step-by-step examples of how to perform single and multi-gene analyses using IIDB via on-line help web pages (under the link 'How To Use IIDB') and through a downloadable tutorial (IIDB Tutorial link).

## Discussion

IIDB is designed to accommodate both experimental data (microarrays, ChIP-chip) and computationally predicted TFBS and genomic annotations. So far, we have only been able to compare the accuracy of our binding site predictions against two datasets. The results are encouraging in both cases. Firstly, there are 80 known TFBS documented within TRANSFAC which map to the IIDB genes. Of these, we capture at least 80%. Second, approximately 50% of the ATF3 ChIP-chip hits coincide with the IIDB predictions, as summarized in Table 2. Thus, the most stringent of the IIDB predictions are sufficiently predictive and small enough in number to allow focused experimental testing. A detailed comparison of the predicted TFBS and experimental data will be presented elsewhere.

**Table 2:** Comparison of IIDB TFBS predictions with ChIP-chip data. Data are presented based on the genome annotations available from both NCBI and ENSEMBL. Note that the annotations differ in the number of predicted genes.

	***Using NCBI coordinates***				***Using ENSEMBL coordinates***			
**Promoter region mapped †**	1000	2000	1000	2000	1000	2000	1000	2000

Number of genes	1151	1151	1151	1151	1935	1935	1935	1935
Unique ATF3 ChIP-chip hits	978	1761	978	1761	1494	2750	1494	2750
								
Conserved promoter regions containing ATF3 TFBS ◇	833	979	833	979	1329	1550	1329	1550
								
**Percentile threshold **■	**0.05**	**0.05**	**0.01**	**0.01**	**0.05**	**0.05**	**0.01**	**0.01**
								
ATF3-group matrices hits*	792	1187	212	299	1333	2029	337	474
ATF3-group matrices within a ChIP-chip segment	442	664	110	165	710	1031	196	272

%overlap between ChIP-chip data & predictions ◉	**55.8**	**55.9**	**51.8**	**55.2**	**53.2**	**50.8**	**58.1**	**57.4**

Notes: ◇ Conserved regions were mapped from the human data of Xie et.al [14]. ■ Threshold refers to the p-value below which predicted TFBS are considered significant. † Numbers refer to length of promoter annotated, in base pairs upstream of the transcription start site. * Since ATF3 binding sites have a strong overlap with CREB binding sites, we used a combined PWM including three ATF matrices and nine CREB matrices to calculate the ATF3 hits. Overlapping hits were collapsed into one as described in the main text. ◉ ChIP fragments in these experiments were estimated to have an average length of approximately 500 bp to 1 Kbp. To determine the coordinates of a ChIP-chip hit, we estimated the center of gravity of a bound region using a moving average filter, then set the start/end coordinates to be +/- 300 bp.

Unlike other TFBS data and prediction repositories, IIDB is implemented to be specific in that it includes data relating to a specific cell type (macrophages) in a specific strain (C57/BL6) of a specific species (mus musculus). We hope that this specificity will prove useful for the immunology community. IIDB is also structured so that the data it contains will be generally useful to the broader immunology community.

### Future Directions

We plan to regularly update the transcription factor binding site data and related statistics within IIDB. We are in the process of building an extended version of IIDB which will include TRANSFAC matrix scans for the entire mouse genome. We also plan to add further ChIP-chip data for various transcription factors we are currently analyzing. We are committed to regularly updating our gene coordinate system with new mouse genome builds. An email-based feedback and help link is provided on the IIDB homepage, and users are encouraged to provide suggestions to continue to refine the utility of IIDB. In this way, we hope IIDB will continue to grow, both in content, and also in its usefulness to the immunology community.

## Conclusion

The current consensus view is that transcription factor binding site prediction based on PWM sequence scans alone is not sufficiently predictive for most systems biology projects. PWM scans generate very high numbers of false positives and numerous overlapping hits. We have used several TFBS prediction algorithms, multi-species conservation information, data on DNase HS sites, and searches based on TFBS meta patterns to reduce the number of hits and increase the predictive power of TFBS predictions. On the basis of available ChIP-chip data, TFBS predictions available via IIDB appear to have a good chance of being confirmed experimentally. We therefore believe IIDB will make a useful contribution to the immunology research community.

Our database currently includes predicted binding sites on the promoters of over 2000 mouse macrophage and immune-specific genes. Results from IIDB analyses of new genes, or new analyses of existing IIDB genes, can be automatically integrated into IIDB following curation. IIDB will grow with time and usage. We have customized a web-based genome browser to simultaneously display multiple genes with multiple annotations and TFBS predictions. Thus, IIDB can be used by researchers without specific computational expertise to develop novel gene regulatory hypotheses.

## Availability and requirements

Project name: Innate Immune Database (IIBD).

Project home page: 

Operating system(s): Platform independent.

Programming languages: Perl/CGI, Java, MySQL.

Licence: free open-access to database via web-interface.

Restrictions to use by non-academics: none.

## Abbreviations

**bp: **DNA sequence base pairs (similarly, Kbp stands for kilo base pairs, and Kb for kilo bases), **ChIP-chip: **Chromatin Immunoprecipitation followed by microarray-based (chip) global identification of ChIP fragments, **CCA**: Cobalt Clustering Algorithm (developed by C. Battail, A. Rust and H. Bolouri) to identify statistically significant spatial clusters of TFBS, **IIDB: **Innate Immune Database, **HS: **DNase1 Hypersensitive Site, **PWM**: Position Weight Matrix for a transcription factor, **TF: **Transcription Factor, **TFBS**: Transcription Factor Binding Site on DNA, **TLR**: Toll-like receptor

## Authors' contributions

MK designed and implemented the IIDB database. MK and AR jointly implemented the user interface.

AR, VT, CB, BL, and DH developed the statistics and algorithms for the transcription factor binding site predictions.

JCR conducted and coordinated the selection and mapping of genes and loci.

All computational work was carried out under the guidance of HB and IS.

KK, JCR, CR, MG, DZ, CB, and BM performed the ChIP-Chip and microarray experiments under the guidance of AA.

HB and MK drafted the manuscript. HB and AGR revised it with input from the other authors. All authors read and approved the final version of the manuscript.

## Supplementary Material

Additional file 1 **Gene uploading and validation process diagram**. When a user uploads genes for annotation via the web interface the requested genes first pass through an extensive verification process. Only positively identified genes are transmitted to the annotation pipeline. At end of the process a notification is sent to the user detailing the status of his/her request. Click here for file

Additional file 2 **Matrix family mapping schematic**. Matrix hits were collapsed into the same group if the start site of the next hit of the family fell within the first half of length of the previous hit of the family. Only the highest scoring matrix of a family was reported. The other matrix hits in the family are displayed as an attribute of the highest scoring matrix. For matrices without other family members, only the highest scoring matrix was reported if the start site of the next identical matrix fell within the first half of the length of the previous matrix. The scores of the other identical matrices are displayed as an attribute of the highest scoring matrix. Click here for file
