# Supplementary figures and images for: The Innate Immune Database (IIDB)
Source: BMC Immunol. 2008 Mar 5;9:7. doi: 10.1186/1471-2172-9-7 (PMC2268913; doi:10.1186/1471-2172-9-7)

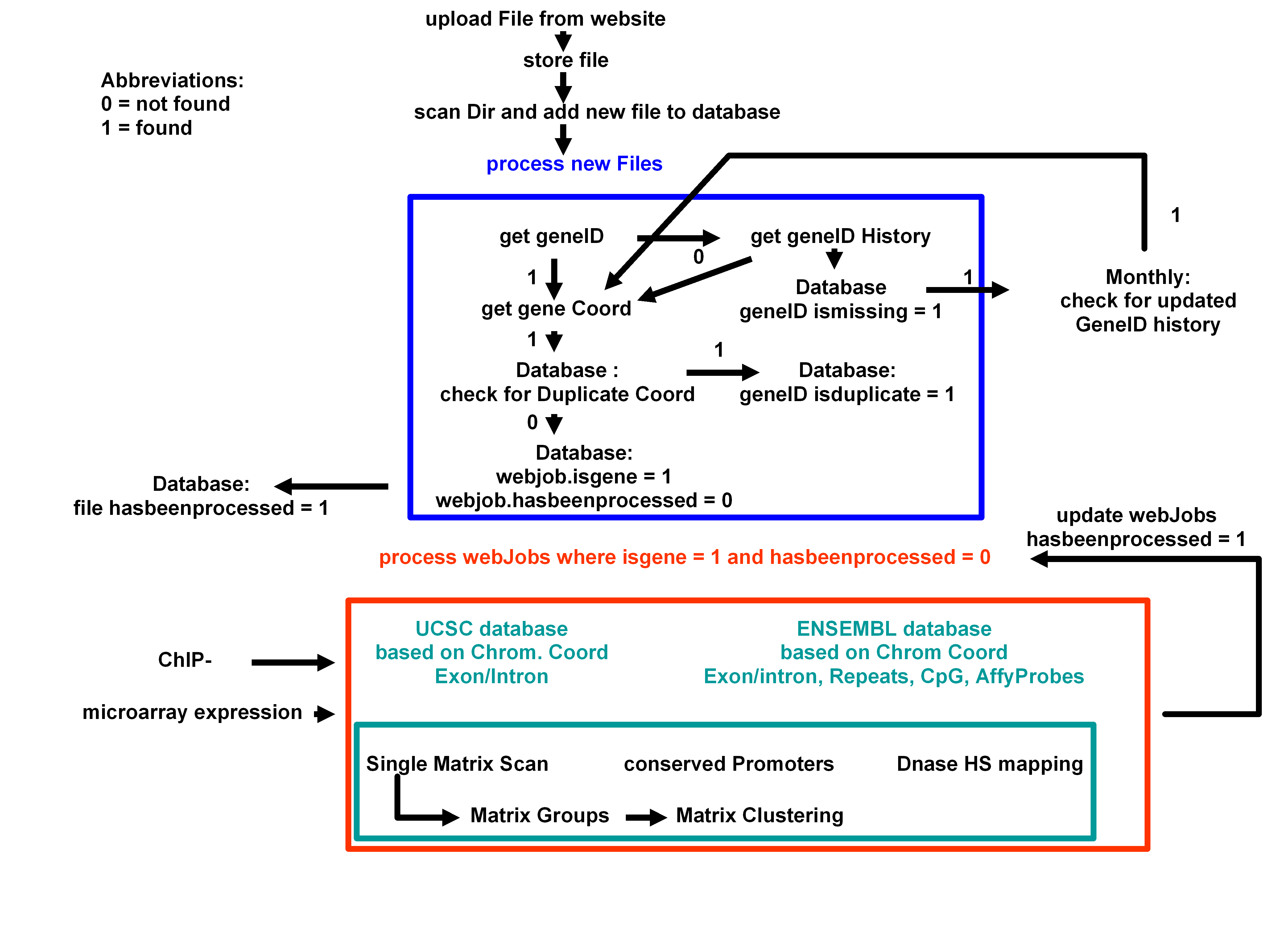

Supplement: Additional file 1 — Gene uploading and validation process diagram. When a user uploads genes for annotation via the web interface the requested genes first pass through an extensive verification process. Only positively identified genes are transmitted to the annotation pipeline. At end of the process a notification is sent to the user detailing the status of his/her request. [file 1471-2172-9-7-S1.tiff]

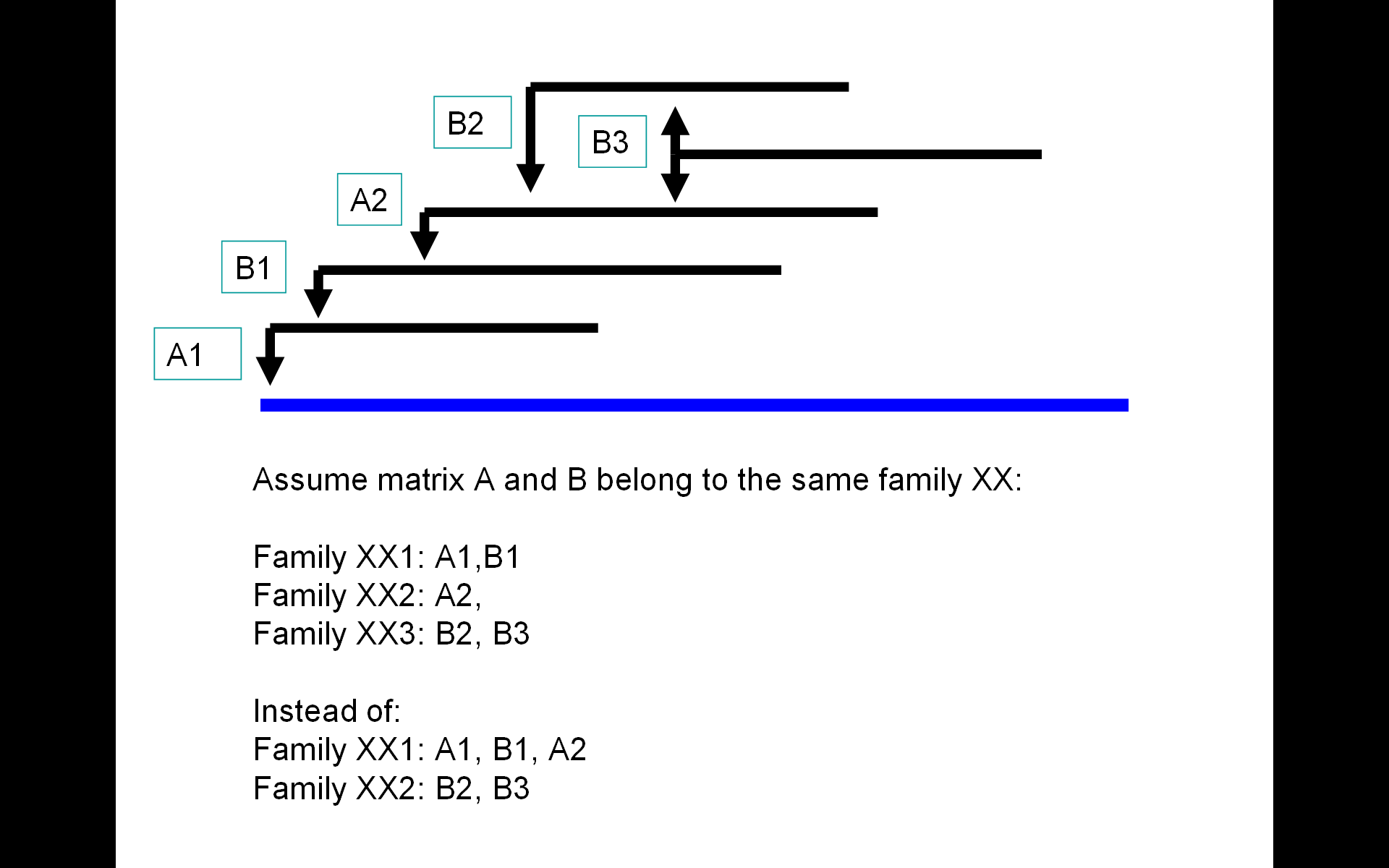

Supplement: Additional file 2 — Matrix family mapping schematic. Matrix hits were collapsed into the same group if the start site of the next hit of the family fell within the first half of length of the previous hit of the family. Only the highest scoring matrix of a family was reported. The other matrix hits in the family are displayed as an attribute of the highest scoring matrix. For matrices without other family members, only the highest scoring matrix was reported if the start site of the next identical matrix fell within the first half of the length of the previous matrix. The scores of the other identical matrices are displayed as an attribute of the highest scoring matrix. [file 1471-2172-9-7-S2.png]
